# Molecular perspective on the American transisthmian species of *Macrobrachium* (Caridea, Palaemonidae)

**DOI:** 10.3897/zookeys.457.6818

**Published:** 2014-11-25

**Authors:** Leonardo G. Pileggi, Natália Rossi, Ingo S. Wehrtmann, Fernando L. Mantelatto

**Affiliations:** 1 Laboratory of Bioecology and Crustacean Systematics (LBSC), Faculty of Philosophy, Science and Letters at Ribeirão Preto (FFCLRP), University of São Paulo (USP), Ribeirão Preto, São Paulo, Brazil; 2 Unidad de Investigación Pesquera y Acuicultura (UNIP) of the Centro de Investigación en Ciencias del Mar y Limnología (CIMAR), Universidad de Costa Rica, 11501-2060 San José, Costa Rica; 3 Museo de Zoología, Escuela de Biología, Universidad de Costa Rica, 11501-2060 San José, Costa Rica.

**Keywords:** Freshwater decapods, genetic variability, molecular phylogeny, Palaemoninae, sibling species

## Abstract

The closure of the Isthmus of Panama (about 3.1 million years ago) separated previously continuous populations and created two groups of extant species, which live now in the Pacific and Atlantic drainage systems. This relatively recent event was a trigger to diversification of various species in the Neotropics, nonetheless there are exemplars that do not show sufficient morphologic variability to separate them by traditional morphological tools. About 60 years ago, some freshwater decapod species with high morphological similarity were separate by previous researchers, based on geographical distribution, in Pacific and Atlantic and considered as “sister species”. However, the complete isolation of these prawns by this geographical barrier is questionable, and it has generated doubts about the status of the following transisthmian pairs of sibling species: *Macrobrachium occidentale* × *Macrobrachium heterochirus*, *Macrobrachium americanum* × *Macrobrachium carcinus*, *Macrobrachium digueti* × *Macrobrachium olfersii*, *Macrobrachium hancocki* × *Macrobrachium crenulatum*, *Macrobrachium tenellum* × *Macrobrachium acanthurus* and *Macrobrachium panamense* × *Macrobrachium amazonicum*. Here we evaluated the relation among these pairs of sibling species in a molecular phylogenetic context. We generated 95 new sequences: 26 sequences of 16S rDNA, 25 of COI mtDNA and 44 of 18S nDNA. In total, 181 sequences were analyzed by maximum likelihood phylogenetic method, including 12 *Macrobrachium* transisthmian species, as well as seven other American *Macrobrachium* species, and two other palaemonids. Our analysis corroborated the morphological proximity of the sibling species. Despite the high degree of morphological similarities and considerable genetic diversification encountered among the transisthmian sister species, our data support the conclusion that all species included in sibling groups studied herein are valid taxonomic entities, but not all pairs of siblings form natural groups.

## Introduction

In the late Pliocene, the closure of the Isthmus of Panama was a trigger to the diversification of many species in the Neotropics. The separation of previously continuous populations created two groups of extant species, which live now in the Atlantic and Pacific drainage systems. This vicariant event opened a unique opportunity for studies on evolution, divergence and speciation processes (Knowlton et al. 1993, Knowlton and Weigt 1998, Lessios 2008). The Central American land bridge is a well-dated biogeographic barrier and is a relatively recent event, about 3.1 million years ago (Keigwin 1978, Coates et al. 1992, Coates and Obando 1996, Anger 2013). Since then, the Atlantic and Paciﬁc marine ecosystems became gradually separated, whereas the gene flow was blocked between organisms on either side.

In spite of the geographic separation, some species are difficult or impossible to distinguish using traditional morphological features, and are thus called “sibling species” (see Knowlton 1993 and references cited therein). These sibling species refer to pairs of species that are genetically closely related, but reproductively isolated (Mayr 1963, Steyskal 1972, Knowlton 1986). Others authors refer to “sibling” as “geminate species” (Jordan 1908, Stillman and Reeb 2001, Marko 2002), in which individuals were separated necessarily by a geographic barrier, and each member of the pair occurs along one coast of the Americas (Lessios 1998, Miura et al. 2010). Other non-morphological features have been used to distinguish these species such as “karyology, hybridization experiments to detect postzygotic incompatibility, distribution patterns, resource use, breeding season, life history and development, mating behavior (including visual, acoustical, and chemical signals), color pattern, and various biochemical characters” (Knowlton 1986). Consequently, a pair of species, reproductively isolated and very similar in morphology, is not necessarily considered as sibling species, and an interdisciplinary approach is necessary to evaluate this conclusion.

Molecular tools have been used to contribute with species delimitation in several cryptic decapods (Schubart et al. 2001a, b, Kitaura et al. 2002, Lai et al. 2010, Pileggi and Mantelatto 2010, Mantelatto et al. 2011, Negri et al. 2012, Torati and Mantelatto 2012). Phylogenies based on molecular data has evidenced probable cases of misidentification of sibling species based on morphology (Lessios 2008, Rossi and Mantelatto 2013). For some freshwater species, the isolation by the closure of the Isthmus of Panama might be questionable, since species of the genus *Macrobrachium* Spence Bate, 1868 can disperse over greater distances than the width of the Isthmus (Steeves et al. 2005, Bauer and Delahoussaye 2008, Bauer 2011) and may also use the Panama Canal as passageway for both sides (Hildebrand 1939, Abele and Kim 1989).

Most studies on decapods sister species focused only in marine species of the genus *Alpheus* Fabricius, 1798 (Knowlton et al. 1993, Knowlton and Weigt 1998, Wehrtmann and Albornoz 2002), while our knowledge of the impact of the Isthmus of Panama on freshwater-invading decapods is extremely limited (Anger 2013). Prawns of the genus *Macrobrachium* are widely distributed in rivers of tropical and subtropical regions with more than 240 recognized species worldwide (De Grave and Fransen 2011). Although its greatest diversity has been found in the Indo-Pacific region, in the Americas there are more than 55 valid species, representing an area of great importance concerning the diversity of the family Palaemonidae (Holthuis 1952, Pileggi and Mantelatto 2012).

The high morphological similarity between some American species led Holthuis (1952) to designate Atlantic and Pacific *Macrobrachium* “sister species”. Until now, morphological similarities between the transisthmian “sibling species” have impeded the identification of the following pairs of species: *Macrobrachium occidentale* × *Macrobrachium heterochirus*, *Macrobrachium americanum* × *Macrobrachium carcinus*, *Macrobrachium digueti* × *Macrobrachium olfersii*, *Macrobrachium hancocki* × *Macrobrachium crenulatum*, *Macrobrachium tenellum* × *Macrobrachium acanthurus* and *Macrobrachium panamense* × *Macrobrachium amazonicum*. These species occur primarily in Central America, with the first species of each pair is found in the Pacific drainage and the second in the Atlantic side. Larvae of these species require saline water (*i.e*., 10–35 ppt) to complete their life cycle, and exhibit other adaptive features, such as extended larval development and amphidromous life histories (Hedgpeth 1949, Bauer and Delahoussaye 2008, Bauer 2011, 2013). Moreover, these prawns show great morphological modifications during ontogenesis, and as other congeneric species they present controversial systematic issues, with high interspecific conservatism and males with intraspecific variation, as found among distinct morphotypes (Holthuis 1952, Moraes-Riodades and Valenti 2004, Pileggi and Mantelatto 2010, Vergamini et al. 2011). Considering the doubt whether the previously indicated species of Central American *Macrobrachium* are sister taxa or not, our study aimed to evaluate in a molecular phylogenetic context the relationships among 12 transisthmian *Macrobrachium* “sibling species” from the Americas in order to assess the validity of their current species level.

## Methods

### Sample collection

Fresh specimens for molecular analysis were obtained from field collections in rivers and estuaries in Brazil, Chile, Venezuela, and Costa Rica (Table 1). The individuals were preserved in 75–90% ethanol and deposited in the Crustacean Collection of the Department of Biology (CCDB), Faculty of Philosophy, Sciences and Letters at Ribeirão Preto (FFCLRP), University of São Paulo (USP), National Institute of Research of Amazônia (INPA) – Brazil, and the Museum of Zoology, School of Biology, University of Costa Rica, Costa Rica (MZUCR). The collections of species conducted in this study complied with current applicable state and federal laws.

**Table 1.** Trans-isthmian species of *Macrobrachium* and other palaemonids used for the phylogenetic analyses, with the respective collection locality, distribution of the species, catalogue number, and genetic database accession numbers at GenBank.

Species	Locality	Distribution	Catalogue Nº	16S	COI	18S
**Sibling species of *Macrobrachium***						
*Macrobrachium acanthurus-1*	Ilha de São Sebastião-SP, Brazil	America-Atlantic	CCDB 2134	HM352445	HM352485	KM101492
*Macrobrachium acanthurus-2*	Guaraqueçaba-PR, Brazil	America-Atlantic	CCDB 2546	HM352444	KM101538	KM101493
*Macrobrachium acanthurus-1*	Puerto Viejo, Costa Rica	America-Atlantic	CCDB 1556	KM101464	KM101537	KM101491
*Macrobrachium acanthurus-2*	Cahuita, Costa Rica	America-Atlantic	CCDB 2901	KM101465	KM101539	KM101494
*Macrobrachium acanthurus-1*	Bocas del Toro, Panama	America-Atlantic	CCDB 3538	KM101467	KM101541	KM101496
*Macrobrachium acanthurus-2*	Panama	America-Atlantic	CCDB 3536	KM101466	KM101540	KM101495
*Macrobrachium tenellum-1*	Puntarenas, Costa Rica	North/Central America-Pacific	MZUCR 1936-002	KM101488	KM101567	KM101534
*Macrobrachium tenellum-2*	Guanacaste, Costa Rica	North/Central America-Pacific	MZUCR 3290-01	KM101489	KM101568	KM101535
*Macrobrachium tenellum*	Oaxaca, Mexico	North/Central America-Pacific	CNCR 24831	KM101487	KM101566	KM101533
*Macrobrachium amazonicum-1*	Santana-AP, Brazil	South/Central America-Atlantic	CCDB 1965	HM352441	HM352486	KM101497
*Macrobrachium amazonicum-2*	Aquidauana-MS, Brazil	South/Central America-Atlantic	CCDB 1970	HM352442	HM352487	-
*Macrobrachium amazonicum-3*	Itacoatiara-AM, Brazil	South/Central America-Atlantic	CCDB 2085	HM352443	HM352488	-
*Macrobrachium amazonicum*	Panama	South/Central America-Atlantic	CNCR 5151	KM101468	KM101542	KM101498
*Macrobrachium panamense-1*	Cerca Camaronera, Costa Rica	Central America-Pacific	MZUCR 2972-01	KM101485	KM101562	KM101528
*Macrobrachium panamense-2*	Río Tempisque, Costa Rica	Central America-Pacific	MZUCR 2971-01	KM101484	KM101561	KM101527
*Macrobrachium panamense-3*	Guanacaste, Costa Rica	Central America-Pacific	MZUCR 3291-01	KM101486	KM101563	KM101529
*Macrobrachium olfersii-1*	Ilha de São Sebastião-SP, Brazil	America-Atlantic	CCDB 2435	HM352459	HM352496	KM101523
*Macrobrachium olfersii-2*	Antonina-PR, Brazil	America-Atlantic	CCDB 2445	HM352458	KM101558	KM101524
*Macrobrachium olfersii*	Isla Margarita, Venezuela	America-Atlantic	CCDB 2446	HM352460	KM101559	KM101525
*Macrobrachium olfersii-1*	Reserva Veragua, Costa Rica	America-Atlantic	CCDB 4873	KM101483	KM101560	KM101526
*Macrobrachium olfersii-2*	Costa Rica (Atlantic)	America-Atlantic	CCDB 2876	JQ805835	JQ805933	JQ805858
*Macrobrachium olfersii-3*	Costa Rica (Atlantic)	America-Atlantic	CCDB 2880	JQ805839	JQ805936	JQ805859
*Macrobrachium digueti-1*	Costa Rica (Pacific)	South/Central America-Pacific	CCDB 2882	JQ805806	JQ805903	JQ805847
*Macrobrachium digueti-2*	Costa Rica (Pacific)	South/Central America-Pacific	CCDB 3091	JQ805807	JQ805904	-
*Macrobrachium digueti-3*	Río Aranjuez, Costa Rica	Central America-Pacific	MZUCR 3292-01	KM101476	KM101551	KM101514
*Macrobrachium digueti*	Mexico	South/Central America-Pacific	CNCR 24811	JQ805808	JQ805906	JQ805849
*Macrobrachium crenulatum-1*	Isla Margarita, Venezuela	South/Central America-Atlantic	CCDB 2124	HM352463	HM352498	KM101512
*Macrobrachium crenulatum-2*	Venezuela	South/Central America-Atlantic	IVIC 123	JQ805801	-	JQ805845
*Macrobrachium crenulatum-1*	Costa Rica	South/Central America-Atlantic	CCDB 2873	JQ805804	JQ805900	JQ805846
*Macrobrachium crenulatum-2*	Costa Rica	South/Central America-Atlantic	CCDB 2877	JQ805800	-	JQ805844
*Macrobrachium crenulatum-3*	Reserva Veragua, Costa Rica	South/Central America-Atlantic	CCDB 4874	KM101475	KM101550	KM101513
*Macrobrachium hancocki-1*	Costa Rica	South/Central America-Pacific	CCDB 3090	JQ805813	JQ805911	-
*Macrobrachium hancocki-2*	Costa Rica	South/Central America-Pacific	CCDB 3092	JQ805814	JQ805912	JQ805851
*Macrobrachium hancocki-3*	Costa Rica	South/Central America-Pacific	CCDB 3757	JQ805821	JQ805920	-
*Macrobrachium hancocki-4*	Costa Rica	South/Central America-Pacific	CCDB 3756	JQ805822	JQ805919	-
*Macrobrachium hancocki*	Panama	South/Central America-Pacific	RMNHD 8810	JQ805817	JQ805915	JQ805852
*Macrobrachium carcinus-1*	Santana-AP, Brazil	America-Atlantic	CCDB 2122	HM352448	HM352490	KM101507
*Macrobrachium carcinus-2*	Ubatuba-SP, Brazil	America-Atlantic	CCDB 2136	HM352449	HM352491	KM101509
*Macrobrachium carcinus*	Isla Margarita, Venezuela	America-Atlantic	CCDB 2123	HM352450	HM352492	KM101508
*Macrobrachium carcinus-1*	Río Suarez, Costa Rica	America-Atlantic	CCDB 2145	HM352452	KM101548	KM101510
*Macrobrachium carcinus-2*	Cahuita, Costa Rica	America-Atlantic	CCDB 4876	KM101474	KM101549	KM101511
*Macrobrachium americanum-1*	Costa Rica	South/Central America-Pacific	CCDB 1731	HM352447	HM352489	KM101499
*Macrobrachium americanum-2*	Río Aranjuez, Costa Rica	South/Central America-Pacific	MZUCR 3292-03	KM101473	KM101547	KM101504
*Macrobrachium americanum-3*	Río Coronado, Costa Rica	South/Central America-Pacific	MZUCR 2963-01	KM101470	KM101544	KM101501
*Macrobrachium americanum-4*	Río Oro, Costa Rica	South/Central America-Pacific	MZUCR 2964-01	KM101471	KM101545	KM101502
*Macrobrachium americanum-5*	Isla Violines, Costa Rica	South/Central America-Pacific	MZUCR 2970-01	KM101472	KM101546	KM101503
*Macrobrachium americanum-6*	Costa Rica	South/Central America-Pacific	CCDB 2883	JQ805797	JQ805899	JQ805843
*Macrobrachium americanum*	Río Cabuya, Panama	South/Central America-Pacific	CCDB 2463	KM101469	KM101543	KM101500
*Macrobrachium heterochirus*	Ilha de São Sebastião-SP, Brazil	South/Central America-Atlantic	CCDB 2137	HM352454	HM352494	KM101515
*Macrobrachium heterochirus-1*	Río Suarez, Costa Rica	South/Central America-Atlantic	CCDB 2899	KM101477	KM101552	KM101516
*Macrobrachium heterochirus-2*	Reserva Veragua, Costa Rica	South/Central America-Atlantic	CCDB 4875	KM101478	KM101553	KM101517
*Macrobrachium heterochirus*	Veracruz, Mexico	South/Central America-Atlantic	Not available	KM101479	KM101554	KM101518
*Macrobrachium occidentale*	Río Aranjuez, Costa Rica	North/Central America-Pacific	MZUCR 3292-02	KM101482	KM101557	KM101522
*Macrobrachium occidentale*	Oaxaca, Mexico	North/Central America-Pacific	CNCR 24838	KM101481	KM101556	KM101521
**Other palaemonids**						
*Macrobrachium borellii*	Buenos Aires, Argentina	South America-Inland waters	UFRGS 3669	HM352426	HM352480	KM101505
*Macrobrachium brasiliense*	Serra Azul-SP, Brazil	South America-Inland waters	CCDB 2135	HM352429	HM352481	KM101506
*Macrobrachium jelskii*	Pereira Barreto-SP, Brazil	South America-Inland waters	CCDB 2129	HM352437	HM352484	KM101519
*Macrobrachium michoacanus*	Oaxaca, Mexico	Mexico-Inland waters	CNCR 24837	KM101480	KM101555	KM101520
*Macrobrachium potiuna*	Eldorado-SP, Brazil	Brazil-Inland waters	CCDB 2131	HM352438	KM101564	KM101530
*Macrobrachium rosenbergii*	Culture, Brazil	Indo-Pacific	CCDB 2139	HM352465	-	KM101531
*Macrobrachium rosenbergii*	Kaohsiung Co., Taiwan	Indo-Pacific	Not informed	-	AB235295	-
*Macrobrachium surinamicum*	Icangui-PA, Brazil	South America-Atlantic	INPA-CR 183	HM352446	KM101565	KM101532
*Cryphiops caementarius*	Region IV, Chile	South America-Pacific	CCDB 1870	HM352453	HM352495	KM101490
*Palaemonetes argentinus*	Parati-RJ, Brazil	South America	CCDB 2011	HM352425	-	KM101536
*Palaemonetes argentinus*	Not informed	South America	Not informed	-	HQ587179	-

Additional material was obtained by donation, visit or loan from distinct worldwide crustacean collections (Table 1). A total of 65 specimens of *Macrobrachium* and three of other genera were analyzed. Almost all sequences were generated in the Laboratory of Bioecology and Crustacean Systematics (LBSC). Some additional comparative sequences were retrieved from GenBank (Table 1). The selection of the other *Macrobrachium* species and genera was based on the phylogeny of Pileggi and Mantelatto (2010), including closely related as well as more phylogenetically distant species. The species identification was based on diagnostic morphological features in accordance with the literature (Holthuis 1952, Villalobos 1969, Melo 2003, Pileggi and Mantelatto 2012).

### DNA extraction, amplification and sequencing

The molecular analysis was based on partial fragments of the 16S rDNA, 18S nDNA and COI mtDNA genes, which have been effective in solving different levels of relationships among decapod species (Schubart et al. 2000, 2001a, b, Porter et al. 2005, Pileggi and Mantelatto 2010, Mantelatto et al. 2011, Vergamini et al. 2011, Carvalho et al. 2013, Rossi and Mantelatto 2013).

DNA extraction, amplification and sequencing protocols followed Pileggi and Mantelatto (2010). Total genomic DNA was extracted from the muscle tissue of walking legs, the chelipeds, or the abdomen. An approximately 530-bp region of the 16S rDNA gene, 560-bp region of the COI gene and 550-bp region of the nuclear 18S gene were amplified from diluted DNA by means of a polymerase chain reaction (PCR) in an Applied Biosystems Veriti 96 Well Thermal Cycler® (thermal cycles: initial denaturing for 5 min at 95 °C; annealing for 40 cycles: 45s at 95 °C, 45s at 48–50 °C, 1 min at 72 °C; final extension 3 min at 72 °C) with the following primers: 16Sar and 16Sbr (Palumbi et al. 1991) for 16S mitochondrial gene; COI-a and COI-f (Palumbi and Benzie 1991) for COI mitochondrial gene; 18Sai and 18Sb3.0 (Whiting et al. 1997) for 18S nuclear gene. PCR products were purified using Sure Clean (Bioline) and sequenced with the ABI Big Dye® Terminator Mix (Applied Biosystems, Carlsbad, CA) in an ABI Prism 3100 Genetic Analyzer® (Applied Biosystems automated sequencer) following Applied Biosystems protocols. All sequences were confirmed by sequencing both strands. A consensus sequence for the two strands was obtained using the computational program BIOEDIT 7.0.5 (Hall 2005). Apart from that, the consensus sequences were blasted on GenBank and compared with our previous sequences. Genetic vouchers generated were deposited in the CCDB under the accession numbers listed in Table 1 or returned with an appropriate label to the original collections.

### Molecular analyses

Sequences were aligned using CLUSTAL W (Thompson et al. 1994) with interface to BIOEDIT with default parameters. Ambiguous alignment regions were removed. Genetic-distance analyzes for the partial sequences of the three markers (16S rDNA, COI mtDNA and 18S nDNA), over sequence pairs between and within groups were conducted in MEGA 5.2 using Kimura-2-parameter model (Tamura et al. 2011). Sequences were analyzed under the Akaike Information Criterion (AIC) (Posada and Buckley 2004) with the program JMODELTEST 2.1.3 (Darriba et al. 2012) to find the best substitution model. The maximum likelihood (ML) analysis was carried out using PAUP 4.0b10 (Swofford 2003). The consistency of topologies was measured by the bootstrap method (1000 replicates), and only confidence values > 50% were reported.

## Results

Our phylogenetic analysis included 12 transisthmian American species of *Macrobrachium*, 7 from other American *Macrobrachium* species, and 2 from palaemonid-related groups. We generated 95 new sequences: 26 mitochondrial 16S sequences, 25 mitochondrial COI sequences, and 44 nuclear 18S sequences. The analysis of the 181 sequences from the three genes produced an alignment of 1.645 bp.

The optimal model for the concatenated data set was the TPM1uf model of sequence evolution (Kimura 1981) plus gamma distributed rate heterogeneity with a significant proportion of invariable sites (TPM1uf +I+G) with the following parameters: assumed nucleotide frequencies A = 0.3028, C = 0.2125, G = 0.1909, T = 0.2937; proportion of invariable sites I = 0.6020; the variable sites followed a gamma distribution, with shape parameter = 0.6700.

**Figure 1.**
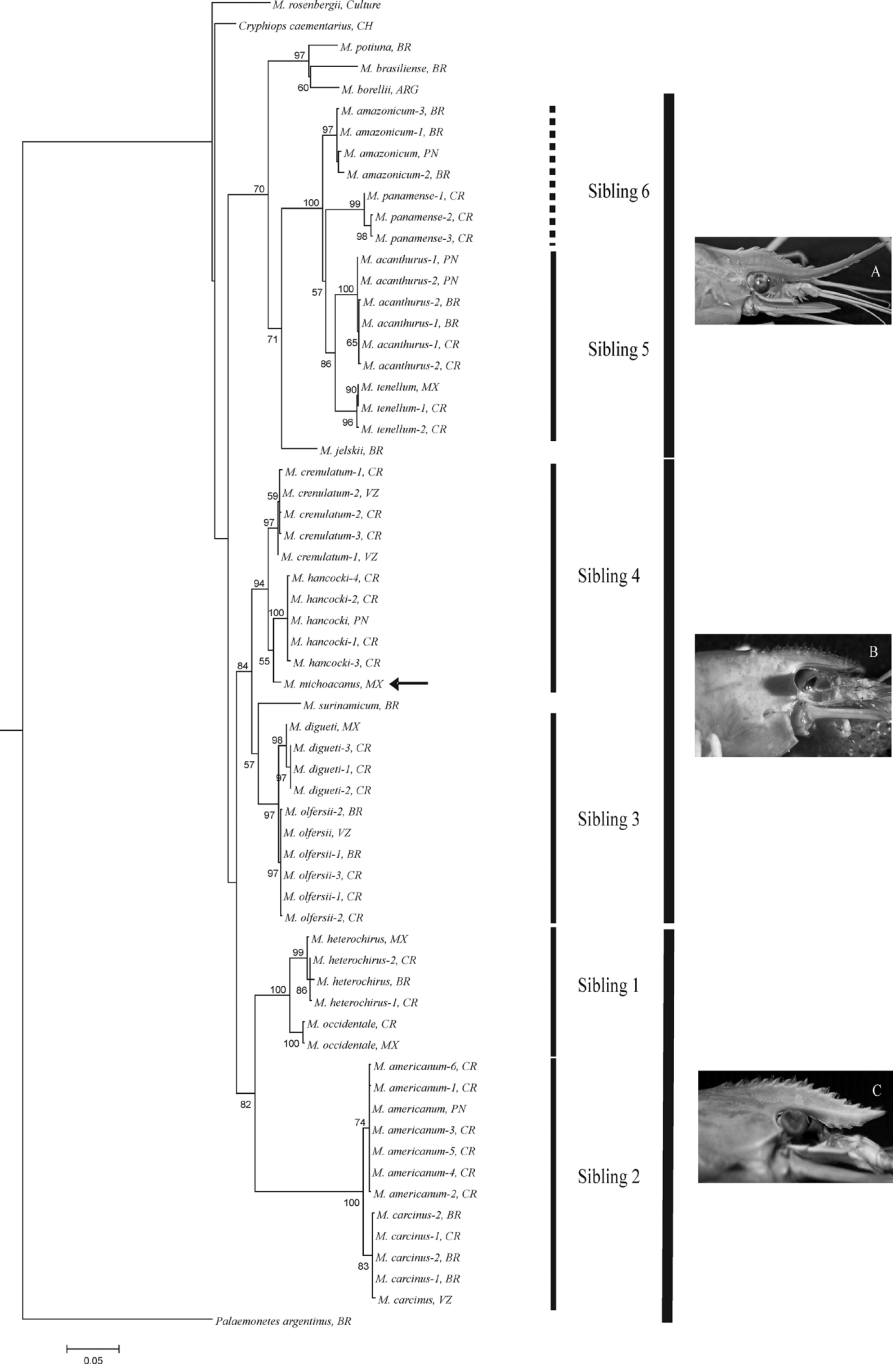
Phylogenetic tree obtained from concatenated maximum likelihood analysis of 16S, COI and 18S sequences for *Macrobrachium* sibling species. Numbers are significance values for 1000 bootstraps; values ≤ 50% are not shown. Abbreviations: ARG: Argentina; BR: Brazil; CH: Chile; CR: Costa Rica; MX: Mexico; PN: Panama; VZ: Venezuela. A: lateral view of the rostrum of *Macrobrachium amazonicum*; B: lateral view of the rostrum of *Macrobrachium olfersii*. C: lateral view of the rostrum of *Macrobrachium carcinus*.

The topology obtained by maximum likelihood from concatenated genes (16S, 18S and COI) analyses confirmed that the transisthmian sibling species (*Macrobrachium heterochirus* × *Macrobrachium occidentale* – Sibling 1, *Macrobrachium carcinus* × *Macrobrachium americanum* – Sibling 2, *Macrobrachium olfersii* × *Macrobrachium digueti* – Sibling 3, *Macrobrachium crenulatum* × *Macrobrachium hancocki* – Sibling 4, and *Macrobrachium acanthurus* × *Macrobrachium tenellum* – Sibling 5) are closely related by well-supported clades (Fig. 1). Sibling 6 (*Macrobrachium amazonicum* × *Macrobrachium panamense*) did not form a separate sister clade despite being phylogenetically close. The position of *Palaemonetes argentinus* showed a stable condition in an external branch. However, the other outgroup (*Cryphiops caementarius*) was maintained within the *Macrobrachium* clade in the phylogeny (Fig. 1). The results did not reveal geographical separation among populations of the same species inside each group (Siblings 1–5). *Macrobrachium michoacanus* (see the arrow in the phylogeny) seems to be close related to *Macrobrachium hancocki* in Sibling 4 group.

The relation among the sibling groups is supported by morphological traits. The species included in Siblings 1 and 2 exhibit similar shapes of the rostrum with the upper margin somewhat arched over the eye and with the apex directed upward (Fig. 1C). Species of the Siblings 3 and 4 with *Macrobrachium michoacanus* and *Macrobrachium surinamicum* show similar rostrum, being almost straight and usually with more than 10 teeth in the upper margin (Fig. 1B). In the same way the species of Siblings 5 and 6 and *Macrobrachium jelskii* have a distinct rostrum, which is elongated, slender, with apex curved upward, with many teeth in the upper and lower margin (Fig. 1A).

In general, distance analyses revealed that the percentage of intraspecific variation was lower than interspecific variation (Table 2). Considering the relation between distinct sibling species, the genetic variability ranged from 4.4% (Sibling 3 × Sibling 4) to 16.9% (Sibling 4 × Sibling 6) for 16S, from 11.3% (Sibling 3 × Sibling 4) to 23.9% (Sibling 2 × Sibling 5) for COI, and from 1.1% (Sibling 5 × Sibling 6) to 11.3% (Sibling 2 × Sibling 5, 6) for 18S (Table 2). Inside each sibling group, the genetic variability varied between 1.5% (Sibling 3) and 8.7% (Sibling 6) for 16S, between 8.3% (Sibling 2) and 16.9% (Sibling 5) for COI, and between 0.0% (Sibling 2, 3) and 1.1% (Sibling 1) for 18S (Table 2).

**Table 2.** Genetic divergence matrix of the 16S and COI mitochondrial genes and 18S nuclear gene among American *Macrobrachium* sibling species obtained by distance analyses using Kimura-2-parameter model. SB: Sibling species. Comparison between the same sibling (bold numbers) comprises interspecific and intraspecific (numbers in parenthesis) analyses.

		SB1	SB2	SB3	SB4	SB5	SB6
**16S**	**SB1**	**0.047–0.046 (0.002–0.013)**					
	**SB2**	0.088–0.103	**0.019–0.028 (0.000–0.006)**				
	**SB3**	0.076–0.093	0.084–0.102	**0.015–0.019 (0.000–0.004)**			
	**SB4**	0.081–0.097	0.076–0.098	0.044–0.065	**0.017–0.021 (0.000–0.011)**		
	**SB5**	0.095–0.136	0.107–0.125	0.115–0.128	0.117–0.136	**0.064–0.069 (0.000–0.004)**	
	**SB6**	0.112–0.146	0.114–0.149	0.115–0.155	0.117–0.169	0.062–0.097	**0.075–0.087 (0.002–0.011)**
**COI**	**SB1**	**0.110–0.128 (0.011–0.061)**					
	**SB2**	0.175–0.233	**0.083–0.122 (0.000–0.038)**				
	**SB3**	0.149–0.179	0.159–0.204	**0.103–0.119 (0.004–0.022)**			
	**SB4**	0.136–0.179	0.168–0.205	0.113–0.168	**0.086–0.109 (0.006–0.091)**		
	**SB5**	0.156–0.197	0.167–0.239	0.147–0.191	0.168–0.209	**0.160–0.169 (0.000–0.022)**	
	**SB6**	0.151–0.180	0.161–0.234	0.143–0.190	0.148–0.196	0.138–0.187	**0.141–0.152 (0.004–0.040)**
**18S**	**SB1**	**0.011 (0.000)**					
	**SB2**	0.097–0.100	**0.000 (0.000)**				
	**SB3**	0.059–0.097	0.097	**0.000 (0.000)**			
	**SB4**	0.044–0.097	0.094–0.097	0.022–0.025	**0.008 (0.000)**		
	**SB5**	0.056–0.059	0.110–0.113	0.053–0.056	0.041–0.047	**0.003 (0.000)**	
	**SB6**	0.056–0.061	0.103–0.113	0.047–0.056	0.039–0.047	0.000–0.011	**0.008 (0.000)**

## Discussion

Over 150 sequences from three different gene regions were used in the present study in order to estimate phylogenetic relationships among freshwater prawns of the genus *Macrobrachium*, which previously were assumed to be transisthmian sibling species. The results revealed that all geminate species studied herein were valid taxonomic entities. Likewise they confirmed the role of the Isthmus of Panama as an effective barrier contributing in the separation of sibling species by the mechanism of allopatric speciation. However, in other cases the separation happened before the closure of the Isthmus probably by the mechanism of sympatric speciation. Our multigenic phylogeny produced consistent groups in most of the pairs of geminate species *i.e.*, sister taxa geographically separated: *Macrobrachium heterochirus* × *Macrobrachium occidentale*, *Macrobrachium carcinus* × *Macrobrachium americanum*, *Macrobrachium olfersii* × *Macrobrachium digueti*, *Macrobrachium crenulatum* × *Macrobrachium hancocki* and *Macrobrachium acanthurus* × *Macrobrachium tenellum*. The constitution of these clades corroborates the morphological proximity of each pair of species as mentioned by Holthuis (1952).

The genetic divergence analyses showed the separation of each sibling group from others, which suggest a consistent relation in comparison with other congeners species (Table 2). Considering that for 16S the divergence in decapods is presumed to be around between 0.6 to 0.9% per Myr (Schubart et al. 2000), we can estimate the divergence time of the sibling species according to the closure of the Isthmus. For Siblings 1 and 5 the time of divergence between the species was approximately from 5.1 to 7.8 and 7.11 to 11.5 Mya, respectively. These estimates predate the closure of the Isthmus, which suggest that the speciation process separated these two species already before the closure. Considering that these amphidromous species are dependent of estuarine water for successful larval development, a sympatric speciation hypothesis seems to be unlikely. However, in these cases the possibility of occurrence of this event is plausible, probably due to environmental changes (Knowlton et al. 1993, Knowlton and Weigt 1998, Morrison et al. 2004). A genetic differentiation could have arisen from a mutational step and the two subpopulations, whose geographic distribution ranges overlap completely, became isolated because both occupy completely different ecological niches (Bush 1994). Analogous events were reported for other crustacean (Malay and Paulay 2009, Jarman et al. 2011). In addition, the estuary can contribute to restriction of the gene flow between the species by distinct selective regimes or habitat fidelity of the species, generating potential speciation in complete or partial isolation (Stanhope et al. 1992, Bilton et al. 2002). Therefore, the sympatric speciation may have occurred in these sibling species by the mechanism of microallopatry (Fitzpatrick et al. 2008). The difficulty in separating *Macrobrachium heterochirus* from *Macrobrachium occidentale*, and *Macrobrachium acanthurus* from *Macrobrachium tenellum* using morphological, ecological and genetic characters (Tables 2, 3, 5), allied with the consistent position in the phylogeny (Fig. 1) provide convincing arguments to consider them as sibling species. The phylogenetic position of Siblings 1 with 2 and Siblings 5 with “6” (here marked between quotes due its artificial position, not characterized as sibling) followed the morphological grouping based on the shape of the rostrum (Fig. 1A, C) indicating that this character is determinant for taxonomic studies.

**Table 3.** Distributional and ecological comparison among each *Macrobrachium* species of sibling pair 1 and 2.

	Sibling 1		Sibling 2	
	*Macrobrachium occidentale*	*Macrobrachium heterochirus*	*Macrobrachium americanum*	*Macrobrachium carcinus*
American slope	Pacific	Atlantic	Pacific	Atlantic
Distribution	Mexico to Panama	USA (Florida) to Brazil (Rio Grande do Sul)	Mexico (Baja California) to Peru	USA (Florida) to Brazil (Rio Grande do Sul)
Habitat	wide range of altitudes (more common in higher elevations of the rivers)		wide range of altitudes (more common in medium and higher courses of the rivers	
Reproduction	require brackish water for reproduction (extended larval development with numerous and small eggs)		require brackish water for reproduction (extended larval development with numerous and small eggs)	
Morphology	very similar and just a few morphological details better seen in adult males are useful characters to separate both species		very similar and present few distinct morphological characters	
References	Holthuis 1952, Mejía-Ortiz et al. 2001, Rocha and Bueno 2004, Almeida et al. 2008, Lara 2009, Villalobos-Hiriart et al. 2010, Lara and Wehrtmann 2011, García-Guerrero et al. 2013, Pileggi et al. 2013		Holthuis 1952, Choudhury 1971, Monaco 1975, Bowles et al. 2000, Mejía-Ortíz et al. 2001, Hernández et al. 2007, Valencia and Campos 2007, Almeida et al. 2008, Lara 2009, Pileggi and Mantelatto 2010, Lara and Wehrtmann 2011, García-Guerrero et al. 2013	

**Table 4.** Distributional and ecological comparison among each *Macrobrachium* species of sibling pair 3 and 4.

	Sibling 3		Sibling 4	
	*Macrobrachium digueti*	*Macrobrachium olfersii*	*Macrobrachium hancocki*	*Macrobrachium crenulatum*
American slope	Pacific	Atlantic	Pacific	Atlantic
Distribution	Mexico (Baja California) to Ecuador	USA (Florida) to Brazil (Rio Grande do Sul)	Costa Rica to Ecuador	West Indies, Panama, Colombia and Venezuela
Habitat	wide range of altitudes (more common in higher elevations of the rivers)		wide range of altitudes (more common in higher elevations of the rivers)	
Ecology	require brackish water for reproduction (extended larval development with numerous and small eggs)		require brackish water for reproduction (extended larval development with numerous and small eggs)	
Morphology	very alike a few characters better seen in adult males are used to separate both species		very similar and can be differentiated using the color pattern, but fixed specimens are difficult to distinguish using only morphological characters	
References	Holthuis 1952, Dugger and Dobkin 1975, Abele and Kim 1989, Wicksten 1989, Rodríguez-Almaraz and Campos 1996, Mejía-Ortiz et al. 2001, Melo 2003, Valencia and Campos 2007, Almeida et al. 2008, Lara 2009, Mejía-Ortiz and Álvarez 2010, Lara and Wehrtmann 2011, Pileggi and Mantelatto 2012, Anger 2013, García-Guerrero et al. 2013, Rossi and Mantelatto 2013		Holthuis 1950, 1952, Wicksten 1989, March et al. 1998, Valencia and Campos 2007, Hein et al. 2011, Lara and Wehrtmann 2011, Anger 2013, García-Guerrero et al. 2013	

**Table 5.** Distributional and ecological comparison among each *Macrobrachium* species of sibling pair 5 and “6”.

	Sibling 5		“Sibling 6”	
	*Macrobrachium tenellum*	*Macrobrachium acanthurus*	*Macrobrachium panamense*	*Macrobrachium amazonicum*
American slope	Pacific	Atlantic	Pacific	Atlantic
Distribution	Mexico (Baja California) to Peru	USA (North Caroline) to Brazil (Rio Grande do Sul)	Honduras to Peru	South American river basins from Venezuela to Argentina
Habitat	wide range of altitudes (more common in median courses of the rivers)		wide range of altitudes (more common in higher elevations of the rivers)	
Ecology	require brackish water for reproduction (extended larval development with numerous and small eggs)		require brackish water for reproduction (extended larval development with numerous and small eggs)	inland (independent of salty water to reproduction) and coastal populations (dependent of salty water to reproduction) (distinct forms of extended larval development with numerous and small eggs)
Morphology	similar and difficult to distinguish		similar, and only few characters are useful features to separate both species	
References	Holthuis 1952, Choudhury 1970, Melo 2003, Hernández et al. 2007, Almeida et al. 2008, Lara and Wehrtmann 2011, Anger 2013, García-Guerrero et al. 2013.		Holthuis 1952, Abele and Kim 1989, Melo 2003, Magalhães et al. 2005, Valencia and Campos 2007, Almeida et al. 2008, Lara 2009, Jara 2010, Vergamini et al. 2011, Anger 2013, Meireles et al. 2013	

The time of divergence between both species of the Sibling 3 was approximately from 1.66 to 3.16 Mya for 16S gene, which supports the efficiency of the barrier in the separation of sibling species by mechanism of allopatric speciation. The morphologically close relation of the “olfersii complex” (see Villalobos 1969 for revision) was corroborated in the phylogeny, where Siblings 3 and 4 form sister groups with the same shape of the rostrum (Fig. 1B), as evidenced in previous molecular results (Rossi and Mantelatto 2013). The entity of the results obtained together with morphological and ecological similarities of *Macrobrachium olfersii* and *Macrobrachium digueti* suggest that both are sibling species, but the inclusion of other species from the *Macrobrachium olfersii* complex in the analysis is necessary to confirm this proposition. Among the sibling species proposed by Holthuis (1952), only one pair (*Macrobrachium surinamicum* × *Macrobrachium transandicum*) was not analyzed in our study due the impossibility to obtain specimens of *Macrobrachium transandicum*. In our phylogeny, *Macrobrachium surinamicum* was included inside the clade of *Macrobrachium olfersii* complex (Villalobos 1969, Rossi and Mantelatto 2013) corresponding to a species with the rostrum almost straight, usually with more than 10 teeth in the upper margin (Fig. 1B).

For Siblings 2 and 4 the time of divergence between the species varied from 2.11 to 4.66 and 1.88 to 3.5 Mya for 16S gene, respectively. These data place them exactly in the range of the closure of the Isthmus, precluding the definition that the separation of the species may have been caused by this vicariant process. Pileggi and Mantelatto (2010) mentioned that *Macrobrachium americanum* could be a synonymous of *Macrobrachium carcinus* based on a single molecular 16S phylogeny. However, and as suggested by the authors, a more extensive sampling of *Macrobrachium americanum* will be necessary to verify this proposition. Our results that include five specimens of *Macrobrachium carcinus* and seven of *Macrobrachium americanum* from distinct localities revealed that both species are sibling species.

In the same way, our data as well as the morphological and ecological similarities evidenced the close relationship between *Macrobrachium crenulatum* and *Macrobrachium hancocki*; however, the addition of data from more specimens and other species from the *Macrobrachium olfersii* complex is necessary to confirm them as sibling species, *i.e.*, sister taxa geographically separated (Rossi and Mantelatto, unpubl. data). Another unpredictable result was the close relation of *Macrobrachium michoacanus* with *Macrobrachium hancocki* (Fig. 1, Sibling 4). With both occurring on the Pacific side, this result may be interpreted as an indication that the relation of phylogenetically closely related congeners living on either side of the Isthmus must be older than the biogeographic barrier separating them (Anger 2013). New diversifications succeeding the closure of the Isthmus occurred at the same side, which can be demonstrated by the higher proximity between these sympatric species than *Macrobrachium hancocki* and *Macrobrachium crenulatum*, the hypothetical Sibling 4. However, analysis of additional material is necessary to verify the phylogenetic position of *Macrobrachium michoacanus*. Following the other sibling species, relationship of the systematic position with the shape of the rostrum was maintained (Fig. 1B–C) supporting the high reliability of this morphological character.

Our results regarding *Macrobrachium amazonicum* × *Macrobrachium panamense* did not confirm a separate sibling group despite the close phylogenetic relation among these species. Our multigenic phylogenetic hypothesis (Fig. 1) indicates *Macrobrachium panamense* as a sister group of the Sibling 5, and *Macrobrachium amazonicum* as a sister species of this group (*Macrobrachium panamense* + Sibling 5). Genetic divergence analyses of the “Sibling 6” pair (8.33 to 14.5 Mya for 16S genes) suggest that the time of their divergence predates the closure of the Isthmus, indicating that both did not share the same ancestral. In addition, the wide geographic distribution of *Macrobrachium amazonicum* in the large South American river basins must be related to geological events driven by the rising Andes along the western portion of these basins (supposedly its native area of occurrence) (see Magalhães et al. 2005 for revision). *Macrobrachium jelskii* as an external clade of Sibling 5 and “Sibling 6” is in agreement with morphological similarities among these species, mainly of the shape of the rostrum (Fig. 1A), despite *Macrobrachium jelskii* being the unique species of the group to present abbreviated larval development. The position of a more external group (*Macrobrachium potiuna*, *Macrobrachium brasiliense*, *Macrobrachium borellii*) with abbreviated larval development in the phylogeny indicates that the ancestral species of this entire group possibly had a life cycle independent of salt water as suggested in previously studies (Murphy and Austin 2005, Pileggi and Mantelatto 2010). *Macrobrachium amazonicum* plays a key role in this puzzle since it presents inland and coastal populations (Vergamini et al. 2011, Meireles et al. 2013), suggesting that the species originated in freshwater environments and entered subsequently in estuarine habitats (Pereira and García 1995, Pileggi and Mantelatto 2010).

Phylogenetic analyses were based on two mitochondrial and one nuclear genes in order to provide a broad spectrum of inference and insights into the evolutionary history of *Macrobrachium* in the Americas. Although the mitochondrial markers may offer strong evidence for genus and species-level relationships, they have high mutation rates, which can cause increasing saturation when older splits are analyzed (Simon et al. 1994, Avise 2004). Therefore, analyses were carried out with sequences from conserved and variable genes to access phylogenetic information across a range of evolutionary time (Crandall et al. 2000). The genes concatenated analysis improve the diversity of evolutionary time, consequently revealed a more consistent phylogeny compared to previous morphological and molecular phylogenetics studies (Pereira 1997, Murphy and Austin 2005, Pileggi and Mantelatto 2010). The inclusion of the member of genus *Cryphiops* within *Macrobrachium* species was maintained in the phylogeny, and raises the question whether *Macrobrachium* is a monophyletic group (Pereira 1997, Murphy and Austin 2005, Pileggi and Mantelatto 2010, Carvalho et al. 2013, Rossi and Mantelatto 2013).

The results of our multidisciplinary approach suggest that species pairs 1-5 refer to siblings, in which each pair of species is difficult to distinguish using traditional morphological characters, although they are genetically distinct, closely related, and reproductively isolated (Mayr 1963, Steyskal 1972, Knowlton 1986). In contrast, our data did not validate “Sibling 6” by molecular analysis, although morphology, ecology, and geographic distribution patterns seem to suggest that they are sibling species (Holthuis 1952). Moreover, the speciation processes of the species of the pairs 2, 3, and 4 seem to have occurred after the rise of the isthmus barrier, probably in Pliocene and Pleistocene by the mechanism of allopatric speciation. However, the isolation of pairs 1 and 5 may have happened before the rise of the isthmus barrier, probably in Miocene by the mechanism of sympatric speciation.

An intriguing case refers to the occurrence of two species (*Macrobrachium hobbsi* and *Macrobrachium olfersii*) on both sides of the Central American land bridge (Anger 2013). The identification of these species may be incorrect or is related to the possibility of a passageway that has started with the opening of the Panama Canal, a scenario that has been already reported (Hildebrand 1939, Abele and Kim 1989). The possible expansion of the distribution range of *Macrobrachium* through the Panama Canal may occur, especially considering the dispersal potential of these amphidromous species (Bauer and Delahoussaye 2008, Bauer 2011, 2013). However the findings of the previous genetic study with *Macrobrachium olfersii* revealed the absence of gene flow between Pacific and Atlantic populations. Moreover, *Macrobrachium digueti* and specimens from Pacific considered as *Macrobrachium olfersii* did not show divergence enough to split them, and the differences were within the range of interspecific values. Therefore, on the Pacific coast only *Macrobrachium digueti* occurs naturally, which is considered, like *Macrobrachium olfersii*, a sibling and cryptic species (Rossi and Mantelatto 2013).

This is the first phylogenetic study using molecular methods devoted entirely to the American transisthmian *Macrobrachium* sister species. Molecular markers confirmed that the Isthmus of Panama is an effective barrier contributing to the separation of sibling freshwater prawns species by the mechanism of allopatric speciation. However, some species seemed to have evolved before the closure of the Isthmus by the mechanism of sympatric speciation. Our phylogenetic analysis revealed consistent groups in most of the studied pairs endorsing the supposed sibling species. In contrast, the position of one pair (*Macrobrachium amazonicum* × *Macrobrachium panamense*) seems to be artificial once they did not share a recent common ancestor. The results presented here contribute to resolution of some doubts about the relationships of geminate American species. Our results support the conclusion that these sibling species are valid taxonomic entities, but not all transisthmian species are the closest living relatives with each other.
